# Resveratrol-induced cytotoxicity in human Burkitt's lymphoma cells is coupled to the unfolded protein response

**DOI:** 10.1186/1471-2407-10-445

**Published:** 2010-08-20

**Authors:** Ying Yan, Yan-Yan Gao, Bao-Qin Liu, Xiao-Fang Niu, Ying Zhuang, Hua-Qin Wang

**Affiliations:** 1 Department of Biochemistry & Molecular Biology, China Medical University, Shenyang 110001, China; 2 Department of Radiotherapy, Shenyang Northern Hospital, Shenyang 110016, China

## Abstract

**Background:**

Resveratrol (RES), a natural phytoalexin found at high levels in grapes and red wine, has been shown to induce anti-proliferation and apoptosis of human cancer cell lines. However, the underlying molecular mechanisms are at present only partially understood.

**Method:**

The effects of RES on activation of unfolded protein responses (UPR) were evaluated using Western blotting, semi-quantitative and real-time RT-PCR. Cell death was evaluated using Annexin V/PI staining and subsequent FACS.

**Results:**

Similar as tunicamycin, treatment with RES lead to the activation of all 3 branches of the UPR, with early splicing of XBP-1 indicative of IRE1 activation, phosphorylation of eIF2α consistent with ER resident kinase (PERK) activation, activating transcription factor 6 (ATF6) splicing, and increase in expression levels of the downstream molecules GRP78/BiP, GRP94 and CHOP/GADD153 in human Burkitt's lymphoma Raji and Daudi cell lines. RES was shown to induce cell death, which could be attenuated by thwarting upregulation of CHOP.

**Conclusions:**

Our data suggest that activation of the apoptotic arm of the UPR and its downstream effector CHOP/GADD153 is involved, at least in part, in RES-induced apoptosis in Burkitt's lymphoma cells.

## Background

There is significant interest in naturally occurring bioactive products that have clinical potential in the prevention and treatment of cancer. Among them is resveratrol (RES), which belongs to a class of defense molecules called phytoalexins and is produced in a wide variety of plants (including grapes, peanuts, and mulberries) in response to stress, injury, UV irradiation, and fungal infection [1]. RES is normally present in many dietary products such as grapes, peanuts, berries and wine [2,3], which is known to affect a broad range of intracellular mediators involved in the initiation, promotion and progression of cancer [3-5]. As an anticancer agent, RES has pleiotropic effects, altering many different signaling pathways, leading to suppression of tumor cell proliferation, adhesion, invasion and metastasis, reduced signs of inflammation and angiogenesis, and induction of apoptosis and differentiation [4,6-13]. Nevertheless, although numerous studies have described intracellular changes leading to cell cycle arrest or apoptosis in response to RES treatment, the effects are often cell type specific [14,15], the precise mechanisms associated with the anti-proliferative and chemopreventive effects of RES have not been well elucidated.

Recently, RES was shown to up-regulate a set of genes involved in endoplasmic reticulum (ER) stress response to unfolded proteins[16]. In addition, induction of CHOP/GADD153, one of the components of the ER stress-mediated apoptosis pathway, was shown to be implicated in RES-induced apoptosis in colon cancer cells [17]. Accordingly, evidence was reported more recently that RES could indeed trigger ER stress-induced cell death in dopaminergic cells[18]. UPR could therefore be a potential mechanism of RES cytotoxicity.

Conditions that disrupt protein folding in the ER, such as a chemical insult or nutrient deprivation, activate stress signaling pathways collectively termed as the unfolded protein response (UPR) [19,20]. The UPR is the major protective and compensatory mechanism enabling stressed cells to survive during ER stress. UPR induction results in both an initial decrease in general protein synthesis, to reduce the influx of nascent proteins into the ER, and increased transcription of ER resident chaperones, folding enzymes, and components of the protein degradative machinery to prevent the aggregation of the accumulating misfolded proteins. The key players in the UPR are well characterized and it is mediated through three ER transmembrane receptors: pancreatic ER kinase (PERK), activating transcription factor 6 (ATF6) and inositol-requiring enzyme 1 (IRE1) [21-23]. In resting cells, all of these ER stress receptors are maintained in an inactive state through their association with the ER chaperone, GRP78 (also called BiP). This interaction is destabilized in the presence of misfolded/unfolded proteins, resulting in the dissociation of GRP78/BiP from PERK, ATF6 and IRE1, thereby initiating the UPR. Initially, the UPR is a pro-survival response enabling the cell to survive reversible environmental stresses. However, if the stress is too severe or lasts for too long, UPR activation eventually leads to cell-cycle arrest and the induction of apoptosis[24-29].

CHOP/GADD153 is a member of CCAAT/enhancer-binding protein family that is induced by ER stress and participates in ER stress-mediated apoptosis [30]. In this study we demonstrate that RES treatment indeed caused the activation of UPR in Raji and Daudi Burkitt's lymphoma cells. Our results demonstrate that a proportion of the ability of RES to kill Burkitt's lymphoma Raji and Daudi cells has been attributed to upregulation of CHOP/GADD153.

## Methods

### Cell culture

Human Raji and Daudi Burkitt's lymphoma cells, human HMy2.CIR B lymphoblast cells were grown as suspension culture in RPMI1640 medium supplemented with 10% FBS. Resveratrol (Sigma-Aldrich, Inc., St. Luis, MO) was dissolved as a 100 mM stock solution in DMSO.

### Viability assay

The in vitro toxicology assay (methyl-thiazol-tetrazolium, MTT based) was performed according to manufacturer's instruction (KeyGEN, Nanjing, China). Cells (1.5 × 10^4 ^cells/100 μl) were incubated in a 96-well plate with different effectors for the times indicated in the figure legends.

### Cell death analysis

For cell death assays, according to the manufacturer's instructions, cells were stained with Annexin V-FITC and propidium iodide (KeyGEN, Nanjing, China) and analyzed by fluorescence-activated cell scanner (FACScan) flow cytometer (Becton Dickinson, Franklin Lakes, NJ).

### RT-PCR detection of unspliced and spliced XBP-1

To determine relative expression levels of XBP-1/XBP-1 s within a sample, the XBP-1 cDNA fragment was amplified with the following pair of primers: 5'-GTTGAGAACCAGGAGTTAAGACAG-3' (forward) and 5'-CAGAGGGTATCTCAAGACTAGG-3' (reverse). A 456-bp PCR product was expected if the XBP-1 cDNA fragment was derived form the unspliced form (that contains the 26-bp intron) and a 430-bp PCR product was expected if the XBP-1 cDNA fragment is derived form the spliced form. The GAPDH fragment was amplified with the following pair of primers: 5'-CTCAGACACCATGGGGAAGGTGA-3' (forward) and 5'-ATGATCTTGAGGCTGTTGTCATA-3' (reverse) to produce a 450-bp fragment of GAPDH. The temperature profile was at 94°C for 2 minutes, followed by 30 cycles of 94°C for 15 seconds, 60°C for 1 minute, and 72°C for 30 seconds. The numbers of PCR amplification cycle of XBP-1 and GAPDH were 35 and 25 respectively. PCR products were run on 2% agarose gels containing ethidium bromide followed by visualization under UV.

### RNA isolation and real-time RT-PCR

Total RNA was isolated from cells using TRIzol reagent (Invitrogen, Carlsbad, CA). Real time PCR analysis was performed in triplication on the ABI 7500 sequence detection system (Applied Biosystems, Foster City, CA) using the SYBR Green PCR Master mix (Applied Biosystems, Warrington, UK). For CHOP, the forward primer was 5'-ATGAGGACCTGCAAGAGGTCC-3' and the reverse was 5'-TCCTCCTCAGTCAGCCAAGC-3'. For GRP78, the forward primer was 5'-GTTCTTGCCGTTCAAGGTGG-3' and reverse was 5'-TGGTACAGTAACAACTGCATG-3'. For GRP94, the forward primer was 5'-TACCCACATCTGCTCCACGTG-3' and reverse was 5'-ACCAAGCTTGATGTTGGTAC-3'. For ATF4, the forward primer was 5'-AAGCCTAGGTCTCTTAGATG-3' and reverse was 5'-TTCCAGGTCATCTATACCCA-3'. For GADD34, the forward primer was 5'-AAGCTCACAGAACCTCTAC-3' and reverse was 5'-GATGTCCACAGAAGAACTTC-3'. For β-actin, the forward primer was 5'-GAGACCTTCAACACCCCAGCC-3' and the reverse was 5'-GGATCTTCATGAGGTAGTCAG-3'. All the reactions were performed in triplicate and normalized using β-actin as control gene.

### Western blot analysis

Cells were lysed in lysis buffer (20 mM Tris-HCl, 150 mM NaCl, 2 mM EDTA, 1% Triton-X100) containing a protease inhibitor cocktail (Sigma-Aldrich, Saint Louis, MO). Cell extract protein amounts were quantified using the BCA protein assay kit. Equivalent amounts of protein (20 μg) were separated using 12% SDS-PAGE and transferred to PVDF membrane (Millipore Corporation, Billerica, MA). Western immunoblotting was performed using primary antibodies against CHOP (Santa Cruz Biotechnology, Santa Cruz, CA), GRP78 (BD Bioscience, San Diego, CA), GRP94 (Abcam, Cambridge, MA), ATF6 (Abcam, Cambridge, MA), eIF2a (Cell Signaling, Danvers, MA), phospho-eIF2a (Ser51) (Cell Signaling, Danvers, MA), phospo-PERK (Thr980) (Cell Signaling, Danvers, MA), Histone H2B (Cell Signaling Technology, Danvers, MA), or GAPDH (Chemicon, Bedford, MA), horseradish peroxidase (HRP)-conjugated anti-rabbit or anti-mouse secondary antibodies (Amersham Biosciences, UK) and ECL solutions (Amersham Biosciences, UK).

### Small interfering RNA (siRNA)

The siRNA sequences used here were as follows: siRNA against CHOP (siCHOP), AAGAACCAGCAGAGGUCACAA and scramble (CCGUAUCGUAAGCAGUACU) that has no homology to any known genes was used as control. In addition, position-mismatched (sequence *underlined*) siCHOP (simutCHOP; AAGAACCAGCAGACCUCACAA) was also used to confirm the specificity of siCHOP. Transfection of siRNA oligonucleotide was performed with Lipofectamine 2000 (Invitrogen, Carlsbad, CA) according to the manufacturer's recommendations. The cells were transfected on three consecutive days, and subsequent treatment was performed 72 h after the first transfection.

### Detection of Ca^2+ ^concentrations

The cytoplasmic level of Ca^2+ ^was determined by flow cytometry (Becton Dickinson FACS Calibur), using Indo 1/AM (Calbiochem, La Jolla, CA). Cells were pretreated with vehicle or BAPTA, a Ca^2+ ^chelator (10 μM) for 3 h before adding 100 μM RES for incubation for 24 h to detect the changes in Ca^2+ ^concentration. The cells were harvested and washed twice, then resuspended in Indo 1/AM (3 μg/ml) and incubated at 37°C for 30 min before being analyzed by flow cytometry.

### Statistics

The statistical significance of the difference was analyzed by ANOVA and post hoc Dunnett's test. Statistical significance was defined as *p *< 0.05. All experiments were repeated three times, and data were expressed as the mean ± SD (standard deviation) from a representative experiment.

## Results

### Rapid phosphorylation of PERK and eIF2α in Raji and Daudi Burkitt's lymphoma cells treated with RES

**Figure 1:**
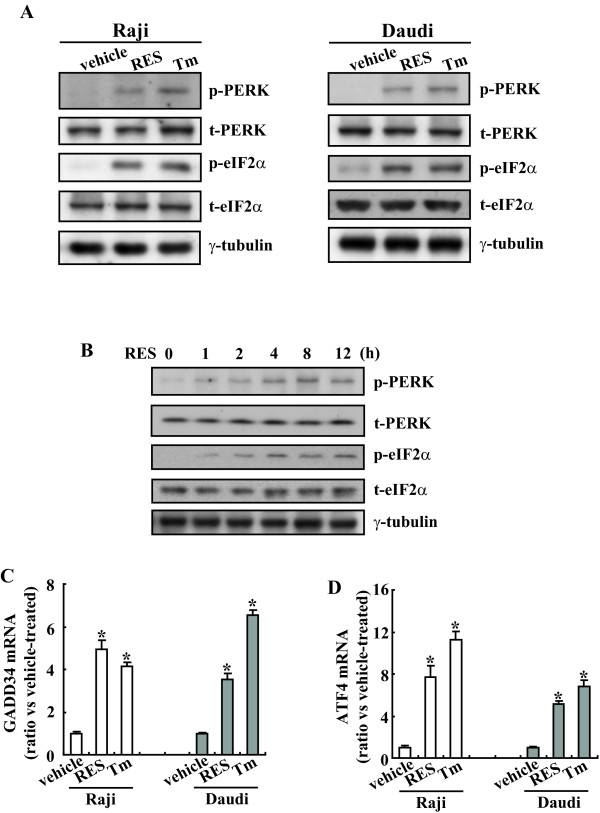
**Activation of PERK and eIF2α signal pathway by RES in Raji and Daudi cells**. A, Raji and Daudi cells were incubated with resveratrol (RES; 100 μM) or tunicamycin (Tm; 10 μg/ml) used as a positive control for 8 h and cell lysates were subjected to Western blotting analysis. B, Raji cells were treated with 100 μM of RES for the indicated time, and Western blotting analysis was performed. C, Raji and Daudi cells were incubated with 100 μM of RES or 10 μg/ml of Tm for 8 h and GADD34 mRNA levels were investigated using real-time PCR. D, Cells were treated as C and ATF4 mRNA levels were analyzed. *, *P *< 0.01.

PERK plays a particularly important role in mediating the global cellular response to ER stress. ER stress induces a PERK-dependent phosphorylation of the a subunit of eukaryotic initiation factor 2α (eIF2α), which leads to a generalized inhibition of translation to reduce the client protein load in the ER[31]. To determine whether this branch of UPR was activated in Burkitt's lymphoma cells treated with RES, we examined the levels of phosphorylated PERK and eIF2α in cells treated with RES. Tunicamycin, a classical ER stress-inducing agent, was simultaneously used to treat Raji and Daudi cells as a positive control. Treatment of Raji and Daudi cells with RES for 8 h caused a marked increase in the phosphorylation of PERK and its direct intracellular substrate, eIF2α (Figure 1A). The phosphorylation of PERK and eIF2α was not associated with an increase in the abundance of these two proteins, suggesting that RES only causes an increase in the fraction of each of these two proteins to become phosphorylated. The time course of the phosphorylation of PERK and eIF2α was then investigated in Raji leukemia cells. PERK phosphorylation was detected at 1 h and steady state of phosphorylation was reached at 4 h after RES exposure (Figure 1B). A similar time course for phosphorylation of eIF2α was observed (Figure 1B). Because IF2a phosphorylation subsequently induces ATF4 and GADD34[32], we next examined the induction of GADD34 and ATF4 mRNA after RES treatment by real-time PCR. RES, as well as tunicamycin, induced GADD34 (Figure 1C) and ATF4 (Figure 1D) mRNA in Raji and Daudi cells. These results indicate that RES induces a pathway initiated by phosphorylation of eIF2α and followed by the upregulation of GADD34 and ATF4.

### Stimulation of XBP-1 splicing by RES in Raji and Daudi cells

**Figure 2:**
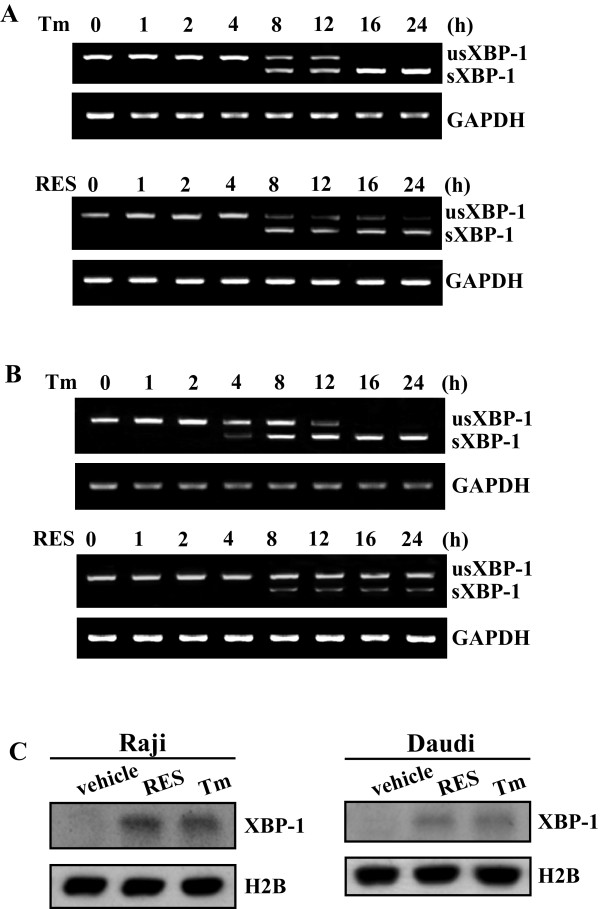
**Stimulation of XBP-1 splicing in Raji and Daudi cells treated with RES**. Raji (A) and Daudi (B) cells were incubated with 100 μM of RES for the indicated time, total RNA was extracted form the cells and the mRNA of XBP-1 was detected and analyzed by RT-PCR. The RT-PCR products were analyzed by agarose gel electrophoresis. GAPDH was used as the internal control. C, XBP-1 expression in nuclear lysates was analyzed using Western blot. The cells were treated with either 100 μM of RES or 10 μg/ml of Tm for 8 h. Antibody against histone H2B was used as loading control.

ER stress also induces activation of the IRE1α endonuclease, which causes the unconventional splicing of XBP-1 mRNA (encoding a transcription factor) in the cytoplasm. The spliced form of XBP-1 mRNA acts as a transcription factor which induced the expression of ER-resident molecular chaperons during ER stress [31]. To evaluate the possible role in induction of IRE1α/XBP-1 pathway by RES, total RNA was extracted from Raji and Daudi cells treated with RES for various time intervals and the XBP-1 was examined by RT-PCR with the primers described in materials and methods. Two binds of 456 bp and 430 bp with 26 bp difference are expected to be amplified, representing the spliced and unspliced mRNA of XBP-1, respectively. Only one XBP-1 cDNA fragment corresponding to the unspliced XBP-1 mRNA was detected in Raji cells after 4 h of RES treatment (Figure 2A). However, an additional XBP-1 cDNA fragment, corresponding to the spliced XBP-1 mRNA was formed as incubation was continued further (8 h and beyond) (Figure 2A). Although the early effects (within 12 h) on XBP-1 splicing after exposure to RES were similar to that seen with tunicamycin, prolonged incubation (up to 24 h) failed to induce further splicing (Figure 2A). A similar time course of generation of the spliced XBP-1 mRNA could be observed when Daudi cells were treated with RES (Figure 2B). The transcription factor protein XBP-1, which is translated from spliced XBP-1 mRNAs, contains a nuclear localization signal and a transcriptional activation domain and activates the transcription of target genes in the nucleus. We then analyzed XBP-1 expression in the nuclear using Western blot analysis. Similar like tunicamycin, RES increased XBP-1 expression both in Raji and in Daudi cells (Figure 2C).

### Activation of ATF6 signaling pathway by RES in Raji and Daudi Burkitt's lymphoma cells

**Figure 3:**
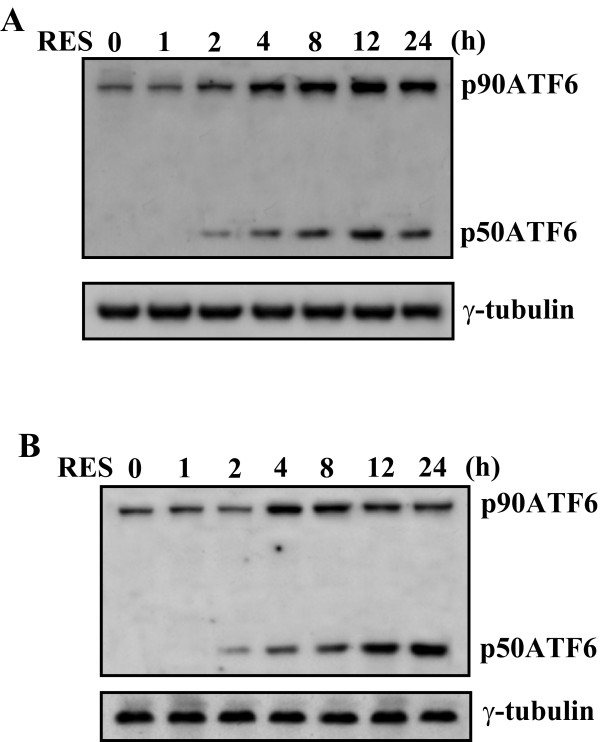
**Increase in the protein levels of 90-kDa ATF6 (p90ATF6), and 50-kDa ATF6 (p50ATF6) in Raji and Daudi cells treated with RES**. Raji (A) and Daudi (B) cells were incubated with or without 100 μM of RES. At the time intervals indicated, the cell lysates were subjected to Western blotting analysis.

In addition of eIF2α phosphorylation and XBP-1 splicing, ER stress activates the ATF6-dependent pathway that subsequently induces the expression of many genes containing the ERSE in the promoter regions, including GRP78/BiP, calnexin, calreticulin, and XBP-1 [33,34]. ATF6 is an integral membrane protein that is found at the ER, upon ER stress, ATF6 is converted from a 90-kDa protein (p90ATF6) to a 50-kDa protein (p50ATF6) transcription factor, resulting in nuclear translocation[35]. Thus, we investigated whether RES induces p50ATF6 by Western blot. In the Raji cells, treatment with RES led to an increase in the protein levels of both the 90-kDa and the 50-kDa ATF6 after 2 h of stimulation (Figure 3A). Similarly, in the Daudi cells, the protein level of the 90-kDa and 50-kDa ATF6 substantially increased with a peak at 4-8 h after addition of RES (Figure 3B).

### Induction of downstream effectors of UPR, GRP78/BiP and CHOP/GADD153 by RES in Raji and Daudi cells

**Figure 4:**
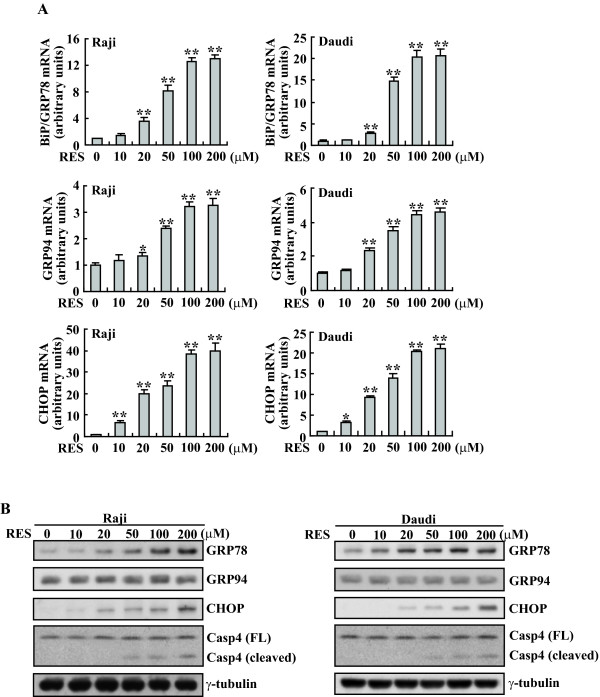
**Induction of BiP/GRP78 and GADD153/CHOP expression by RES in Raji and Daudi cells**. A, Raji and Daudi cells were treated with different concentration of RES for 8 h and real-time PCR was performed. B, Raji and Daudi cells were treated with the indicated concentration of RES for 24 h and cell lysates were subjected to Western blotting analysis. *, *P *< 0.05; **, *P *< 0.01.

GRP78/BiP is the key chaperone for folding and maturation of protein in ER and its upregulation is the usual marker of ER stress. The real-time PCR analyses indicated that GRP78/BiP in Raji and Daudi cells increased by approximately 12 and 20 fold after 100 μM of RES exposure, respectively (Figure 4A). Western blot analysis confirmed that GRP78/BiP protein levels were also significantly increased in response to treatment with RES (Figure 4B). Compared with GRP78/Bip, lower induction of GRP94 were observed in response to RES, with only 3- to 5-fold changes of mRNA transcript being detected with 100 μM of RES treatment (Figure 4A), GRP94 protein levels demonstrated unaltered or marginal increase throughout this dose range (Figure 4B). Another usual marker of ER stress, CHOP/GADD153, which is an apoptotic effector protein situating functionally downstream of the UPR signaling pathways, was also investigated. The basal level of CHOP/GADD153 was extremely low, on exposure to RES Raji and Daudi cells depicted a marked increase in the concentrations of CHOP mRNA (Figure 4A) and protein (Figure 4B) in a dose-dependent manner. In addition, RES treatment also caused dose-dependent cleavage of caspase-4 (Figure 4B), which has been shown to be predominantly located to the outer membrane of the ER, and to play important roles in ER stress-induced apoptosis[31,36,37].

### Involvement of ER stress in RES-induced cell death

**Figure 5:**
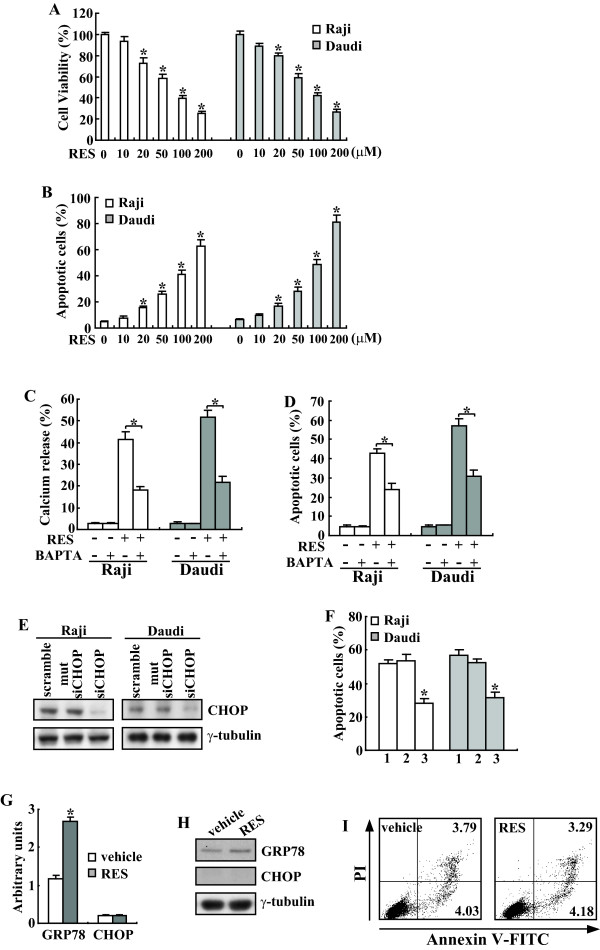
**Prohibition of CHOP induction attenuates RES-induced apoptosis in Raji and Daudi cells**. Raji and Daudi cells were treated with the indicated concentration of RES for 24 h and cell viability (A) and apoptotic cells (B) were analyzed using MTT and FACS, respectively. C, Cells were pretreated with vehicle or BAPTA for 3 h, then treated with RES for 24 h and cytosolic calcium was analyzed using flow cytometry. D, Cells were treated as C, and apoptotic cells were analyzed using Annexin V/PI staining followed by flow cytometry. E, Cells were transfected with scramble, mutant siRNA against CHOP (mutsiCHOP) or siRNA against CHOP (siCHOP) on three consecutive days, treated with 100 μM of RES for 24 h and cell lysates were subjected to Western blotting analysis. F, Cells were treated as C and apoptotic cells were analyzed using FACS. G, HMyc2.CIR cells were treated with indicated concentration of RES for 8 h and real-time PCR was performed. H, HMyc2.CIR cells were treated with indicated concentration of RES for 24 h and Western blot analysis was performed. I, HMyc2.CIR cells were treated with 100 μM of RES for 24 h, apoptosis of cells was analyzed using flow cytometry. *, *P *< 0.01.

We therefore examined if RES might actually cause the activation of UPR which in turn caused cell death. MTT analysis showed that treatment of Daudi and Raji cells with RES greatly reduced cell proliferation and viability in a dose-dependent manner (Figure 5A). This inhibitory effect became apparent at a concentration of 20 μM RES (Figure 5A). To determine whether the decrease in cell viability was attributable to apoptosis, cells were stained with FITC conjugated Annexin V plus PI and evaluated by FACS. Both Daudi and Raji cells underwent dose-dependent apoptotic cell death in response to RES (Figure 5B).

Since the role of cytosolic Ca^2+ ^as proapoptotic messenger involved in ER stress-mediated apoptosis has been ascertained[38], we investigated whether RES treatment affects cytosolic Ca^2+^. Addition of RES was found to significantly increase cytosolic Ca^2+ ^(Figure 5C). Treatment of cells with the intracellular Ca^2+ ^chelator BAPTA (10 μM for 3 h) prior to RES treatment (24 h), caused an effective chelation of cytosolic Ca^2+ ^(Figure 5C). Importantly, pretreatment with BAPTA also mitigated apoptosis induced by RES (Figure 5D).

CHOP/GADD153 is one of the components of the ER stress-mediated apoptosis pathway. To further investigate the potential involvement in ER stress-mediated apoptosis by RES, we examined whether induction of CHOP/GADD153 is critical to induce RES-mediated Burkitt's lymphoma cell death by siRNA duplex against CHOP mRNA. Daudi and Raji cells were transfected with the indicated siRNA were treated with vehicle or RES. Western blot analysis demonstrated that siRNA against CHOP effectively prohibited its upregulation mediated by RES (Figure 5E). Importantly, under these conditions, apoptosis induced by RES was significantly attenuated in Daudi and Raji cells transfected with CHOP siRNA when compared with scramble siRNA or siRNA against mutant CHOP-transfected cells (Figure 5F).

Next we investigated whether RES caused normal lymphocyte death via induction of ER stress. Real-time PCR indicated that in normal B lymphoblast HMy2.CIR cells, RES increased the GRP78/Bip transcript with about 2 folds upon exposure to 100 μM of RES (Figure 5G). It should be noted that same concentration of RES resulted in 12 and 20 folds of GRP78/Bip induction in Raji and Daudi cells, respectively (4A). Importantly, no obvious induction of CHOP was observed in RES treated HMy2.CIR cells (5G). Consistent with real-time PCR, Western blot demonstrated that GRP78/Bip protein levels were increased upon RES exposure, whereas, CHOP proteins were undetectable in HMy2.CIR cells with or without RES treatment (Figure 5H). Flow cytometry demonstrated that 100 μM of RES had no obvious effects on apoptosis of HMy2.CIR cells (Figure 5I).

## Discussion

The use of nontoxic chemical substances is considered a promising alternative strategy for the treatment of human cancer. In recent years, many natural or dietary substances have been shown to inhibit experimental carcinogenesis[39]. In this regard, RES, a phytoalexin found in grapes and peanuts hat has shown promise as a novel chemotherapeutic agent, which exerts a wide array of biological effects, including anti-inflammatory, anti-proliferative and potential chemopreventive activity against human cancer[40]. Moreover, RES has been shown to suppress the growth of transformed cells also through induction of apoptosis[9,41,42]. Over the past decade, RES has emerged as one of the most promising naturally occurring compound with immense therapeutic potential. However, unlike other commonly occurring natural or synthetic drugs, the precise effect and mode of action of RES has remained enigmatic. In this study we tried to establish the pro-apoptotic role of RES in Burkitt's lymphoma cells and to decipher the mechanisms underlying this action. We showed that treatment of Daudi and Raji Burkitt's lymphoma cells with RES was able to induce ER stress and activated all 3 branches of the UPR. It was interesting to note that both the full-length and cleaved ATF6 increased upon RES exposure. Full-length, as well as cleaved ATF6 was also reported to be increased in cells treated with 4HPR[43]. Since lack of information on the metabolism of these two proteins at the present, the underlying mechanisms remain to be clarified in the future.

The mechanism of ER stress and the unfolded protein response is primarily a cell protective mechanism [44,45], resulting in transient induction of cell cycle arrest and accumulation of molecular chaperons such as GRP78/BiP to bind and recover unfolded proteins. However, it has repeatedly been described that prolonged exposure of cells to either ER stress can induce a switch from cell survival to apoptosis, and the cell protective function of these mechanisms appears to be only a timely restricted protection[44,46]. The induction of GADD153/CHOP, synthesized as a downstream component upon the activation of PERK/eIF2a pathway, may be related to the cell death-mediating effect of ER stress. GADD153/CHOP is a proapoptotic protein that is able to downregulate the expression of Bcl-2, and to upregulate the expression of some proapoptotic members of the Bcl-2 family[47,48]. Overexpression of GADD153/CHOP has been reported to lead to cell cycle arrest and apoptosis, which are believed to be important targets for cancer drug development[30]. In the current study, we found that RES exposure induced apoptotic executor GADD153/CHOP expression in Raji and Daudi cells. Furthermore, we demonstrated that prohibition of GADD153/CHOP induction attenuated RES-induced cytotoxicity in Raji and Daudi cells, suggesting that induction of apoptotic branches of UPR might be implicated in RES-mediated cell cytotoxicity. The activation of UPR by RES was reported in dopaminergic cells recently [18] and data described in this study was provided further insights as to how UPR might be involved in the cytotoxic action of RES in Burkitt's lymphoma cells.

ER is a principal site for protein synthesis and modification prior to directing protein delivery to other organelles and its proper functioning is essential for cell survival. Any external or internal factors, such as calcium store depletion, inhibition of glycosylation, reduction of disulfide bonds, et al., that impinge on ER structure and function will ultimately result in accumulation of unfoled or misfolded proteins, leading to ER stress[20,22]. RES might activate ER stress responses via different mechanisms. For instance, although preferentially functioning as an antioxidant, RES paradoxically has a propensity to stimulate formation of reactive oxygen species (ROS) in some cells [49,50], which can cause oxidation of nascent proteins, thus leading to misfolded proteins and ER stress. It has also been reported that RES inhibits 20 S proteasomal activity[18], which can cause accumulation of misfolded or unfolded proteins and ER stress. In addition, resveratrol mimics the situation of calorie restriction and ATP deficiency[51], which can hinder proper folding of nascent proteins. Furthermore, red wine polyphenol compounds is reported to increase intracellular calcium[50], suggesting that RES possibly cause ER stress through regulation of calcium store in ER. In the current study, pretreatment with BAPTA for 3 hours dramatically reduced RES-induced apoptosis. Apparently, Ca2+ plays an important role in RES-induced apoptosis in Burkitt's lymphoma cells. Further deciphering the mechanisms by which RES leads to ER stress in details, might potentiate the combinational treatment using RES and other inducers of ER stress to combat with malignancies.

## Conclusions

RES activates all three branches of UPR in Burkitt's lymphoma cells. In addition, activation of the apoptotic arm of the UPR and its downstream effector CHOP/GADD153 is involved, at least in part, in RES-induced apoptosis in Burkitt's lymphoma cells.

## Competing interests

The authors declare that have no competing interests.

## Authors' contributions

YY carried out the molecular genetic studies, cell culture, and participated in the data analysis. YYG carried out the DNA cloning and flow cytometry. BQL participated in real-time PCR and cell culture. XFN participated in the DNA cloning and cell culture. YZ participated in flow cytometry and MTT assay. HQW conceived of the study, and participated in manuscript drafting and coordinate. All authors read and approved the final manuscript.

## Pre-publication history

The pre-publication history for this paper can be accessed here:

http://www.biomedcentral.com/1471-2407/10/445/prepub
